# Testosterone status and bone mineral density in Finnish male endurance athletes: Baseline associations and 1‐year follow‐up

**DOI:** 10.14814/phy2.70972

**Published:** 2026-06-10

**Authors:** Adam Wagner, Katja Mjøsund, Riina Komonen, Anthony C. Hackney, Johanna K. Ihalainen

**Affiliations:** ^1^ Department of Sport Performance and Exercise Testing, Faculty of Sport Studies Masaryk University Brno Czech Republic; ^2^ University of Jyväskylä, Faculty of Sport and Health Sciences Jyväskylä Finland; ^3^ National Olympic Training Centre Helsinki Finland; ^4^ Paavo Nurmi Centre and Unit for Health and Physical Activity University of Turku Turku Varsinais‐Suomi Finland; ^5^ Finnish Institute of High‐Performance Sport KIHU Jyväskylä Finland; ^6^ Department of Exercise & Sport Science—Department of Nutrition University of North Carolina Chapel Hill North Carolina USA

**Keywords:** bone mineral density, endurance athletes, IGF‐1, testosterone

## Abstract

This study examined whether low‐normal serum total testosterone (*T*) is associated with bone mineral density (BMD) or bone stress injuries (BSI) in male endurance athletes. Baseline data from 46 national‐ to international‐level Finnish athletes (age 24.8 ± 3.6 years; biathlon, cross‐country skiing, orienteering, triathlon, Nordic combined, endurance running) were analyzed; 31 completed 1‐year follow‐up. Participants were categorized into low *T* (lowest quartile: 11.8 ± 1.8 nmol/L) and normal *T* groups (17.9 ± 3.1 nmol/L; *p* < 0.001). At baseline, BMD and *Z*‐scores did not differ at any site (all *p* > 0.5), and BSI history prevalence was similar in low *T* (41.7%) and normal *T* groups (29.4%). The low *T* group had lower IGF‐1 (*p* = 0.031) but comparable cortisol and insulin. During follow‐up, repeated‐measures analysis showed a significant group‐by‐time interaction for *T* (*p* = 0.001), whereas 1‐year changes in BMD and *Z*‐scores did not differ between groups. Regression models showed that increased IGF‐1 independently predicted improved total body BMD (*p* = 0.01). In conclusion, low‐normal *T* in elite male endurance athletes was not associated with compromised bone health over 1 year and appeared largely reversible in the follow‐up subset.

## 1 INTRODUCTION

Endurance training imposes sustained physiological demands on multiple systems, including those governing endocrine function and skeletal integrity. Accumulating evidence indicates that both male and female endurance athletes with signs of hormonal and nutritional imbalance are at greater risk of impaired bone health and bone stress injuries (Barrack et al., 2014; Kraus et al., 2019). While hormonal disturbances and impaired skeletal health have been extensively documented in female athletes, male endurance athletes may also experience subclinical reductions in testosterone (*T*) and bone mineral density (BMD), particularly under conditions of prolonged high training loads and low energy availability (LEA) (Mountjoy et al., 2023; Popp et al., 2022). Relative Energy Deficiency in Sport (REDs) refers to the health and performance consequences of problematic low energy availability in athletes, with possible multisystem effects including endocrine function and bone (Mountjoy et al., 2023).

Hypothalamic–pituitary–gonadal (HPG) axis dysfunction has been proposed as one of the key mechanisms linking prolonged energy imbalance to suppressed *T* levels in male athletes (Hackney, 2020). However, circulating *T* concentrations are not static and may vary across days and weeks in response to training load, recovery status, sleep, illness, and short‐term changes in energy availability (Cupka & Sedliak, 2023; Hackney, 2020; Hackney & Hackney, 2005; McGuire et al., 2024). Thus, a single standardized morning blood sample should be interpreted as an indicator of endocrine status at that assessment rather than direct evidence of persistent suppression. Within the REDs framework, problematic LEA can impair gonadotropin‐releasing hormone (GnRH) secretion and lead to suppressed *T* production, often in the context of a broader catabolic or energy‐conservation profile that may include changes in IGF‐1, insulin, cortisol, body composition, recovery, and injury risk (Mountjoy et al., 2023; Popp et al., 2022). In contrast, the Exercise Hypogonadal Male Condition (EHMC) has been described as a more chronic adaptive‐regulatory state in some endurance‐trained men, characterized by persistently reduced *T* without clear clinical symptoms or impaired performance (Hackney, 2020; Hackney & Hackney, 2005). Low or low‐normal *T* alone is therefore not specific to either REDs/LEA or EHMC; these mechanisms may also overlap in individual athletes. Differentiating between transient LEA‐related suppression, nonfunctional overreaching, and a more stable EHMC‐like adaptation is essential when assessing hormonal profiles in this population, particularly in relation to skeletal health (Hackney, 2020; Hooper et al., 2017; Mountjoy et al., 2023).

Despite increasing recognition of these endocrine–skeletal interactions, there remains limited empirical research characterizing how variations in *T* concentrations relate to bone outcomes in male endurance athletes (Hutson et al., 2021; Mountjoy et al., 2023). Bone health is multifactorial and is influenced not only by sex hormones, but also by osteogenic loading history during adolescence and adulthood, sport‐specific impact loading, lean mass, energy and nutrient intake, calcium and vitamin D status, and the broader endocrine milieu (Elliott‐Sale et al., 2018; Haines et al., 2023; Hutson et al., 2021; Popp et al., 2022; Tenforde et al., 2016). Moreover, the clinical and physiological relevance of subclinical *T* suppression in this population, particularly when levels fall within the low‐normal range, remains unclear (Hutson et al., 2021; Shigehara et al., 2021). Notably, reductions in *T* concentrations of 10–40% have been observed in male athletes with LEA, yet these levels often fall within clinically normal ranges, highlighting the limited sensitivity of conventional reference thresholds to detect endocrine disruption in athletic populations (Heikura et al., 2018; Tenforde et al., 2016). Because areal BMD reflects skeletal adaptation over months to years, only small changes may be expected during a 1‐year follow‐up, and baseline hormonal status should be interpreted cautiously as a predictor of bone outcomes. Although several studies have explored cross‐sectional associations between *T* and bone health in male endurance athletes, longitudinal research evaluating whether baseline hormonal profiles predict changes in BMD or injury risk over time is lacking (Haines et al., 2023; Hutson et al., 2021). Addressing these gaps is important to determine whether testosterone levels at the lower end of the clinical reference range represent a functional adaptation, a transient response to training or low energy availability, or a subclinical risk marker for skeletal health.

As such, this study aims to investigate the relationship between *T* status and bone health in national‐ to international‐level Finnish male endurance athletes. Specifically, we sought to test the hypothesis that athletes in the lowest quartile of the normal *T* range might exhibit early signs of compromised bone density compared to their peers with higher *T* levels. Baseline analyses compared BMD, hormonal and metabolic markers, and bone stress injury history between athletes classified by *T* quartiles, with the lowest quartile representing the low *T* group. Additionally, in a subset of athletes, a 1‐year follow‐up evaluated whether 1‐year changes in bone and endocrine parameters differed according to baseline *T* status.

## 2 MATERIALS AND METHODS

### 2.1 Study design and recruitment of participants

We used baseline observational and 1‐year follow‐up data from NoREDS (Athletic Performance and Nutrition), a 3‐year follow‐up study conducted at the University of Jyväskylä, Finland. A total of 391 Finnish male and female athletes competing at the national to international level, aged 16–35 years at baseline, were included and followed up between 2021 and 2024. Athletes were assessed at the beginning (*T*1) and end (*T*2) of their training season for 1 to 3 years. At *T*1, athletes had recently completed their off‐season and were entering the general preparation phase. At *T*2, assessments took place at the end of the specific preparation phase, just before the competition season. For the present analysis, the first eligible *T*1 assessment from male endurance athletes was used for the baseline analysis, and the subsequent *T*1 assessment approximately 1 year later was used for the follow‐up analysis when available.

A total of 46 national‐ to international‐level (tiers 3–5 (McKay et al., 2022)) male endurance athletes were included in the baseline analysis, comprising 7 biathletes, 7 cross‐country skiers, 18 orienteers, 1 triathlete, 6 Nordic combined athletes, and 7 endurance runners. Most participants competed at the international level, whereas some performed at a high level in national competitions and were training to reach the international level. A total of 31 athletes completed the 1‐year follow‐up, including 5 biathletes, 3 cross‐country skiers, 16 orienteers, 1 triathlete, and 6 endurance runners.

The study followed the Declaration of Helsinki and was approved by the Ethics Committee of the University of Jyväskylä (NoREDS 514/13.00.04.00/2021). Participants gave written informed consent before enrolling in the study. All data were pseudonymized before analysis.

### 2.2 Body composition and bone mineral density

The height of the participants was measured using a stadiometer attached to the wall. A scale (Detecto, Knorring, Finland) was used to determine body mass. Body composition and BMD were measured using dual‐energy X‐ray absorptiometry (DXA) (Prodigy, Encore, SP 4.1, version 18, GE Medical Systems, Madison, Wisconsin, USA). A whole‐body scan was performed to assess body composition and total body BMD, followed by site‐specific scans of the lumbar spine (L1–L4) and proximal femur/femoral neck. Participants wore light clothing, were assessed after an overnight fast, and had voided prior to the measurements. All scans were performed on the same scanner by trained research staff following a standardized positioning and analysis protocol for athletes and active populations. Scanner quality‐assurance calibration was performed according to the manufacturer's recommendations before scanning sessions. *Z*‐score values were analyzed according to recommended best practice guidelines (Nana et al., 2015). Clinically low BMD was defined as a *Z*‐score below −2.0 in line with ISCD recommendations; in addition, *Z*‐scores below −1.0 were reported descriptively as a REDs/Triad‐relevant indicator of potential bone‐health concern.

### 2.3 Blood samples

Fasting venous blood samples were collected from the antecubital vein while participants were in a supine position between 7:00 and 10:00 am after an overnight fast of at least 8 h. Whole blood was allowed to clot for 30 min at room temperature, then centrifuged at 2200 × g for 10 min before aliquoting and storage at −80°C.

Serum total testosterone (*T*), IGF‐1, cortisol, SHBG, and insulin concentrations were measured using the IMMULITE 2000 XPi analyzer (Siemens Healthcare Diagnostics, UK), following the manufacturer's instructions. The IMMULITE assay catalog/reference numbers and kit lots were: total testosterone, L2KTW2 (kit lot 668); IGF‐1, L2KGF2 (kit lot 298); cortisol, L2KCO2 (kit lot 620); SHBG, L2KSH2 (kit lot 431); and insulin, L2KIN2 (kit lot 532). The measurements of serum 25(OH)D were performed using electrochemiluminescence immunoassays (ECLIA).

The interassay coefficient of variation (CV), determined in our laboratory, and analytical sensitivities for hormones were as follows: *T* 6.7% and 0.5 nmol·L^−1^, IGF‐1 7.7% and 2.6 nmol·L^−1^, cortisol 8.2% and 5.5 nmol·L^−1^, SHBG 6.3% and 0.2 nmol·L^−1^, and insulin 11% and 0.49 IU/mL^−1^.

Participants were divided into low and normal *T* groups based on the lowest quartile of baseline serum total testosterone concentration. This approach was chosen because, although *T* levels may be reduced in endurance‐trained athletes, values often remain within the normal clinical range established for the general population. Using a statistical threshold based on quartiles therefore allows for the assessment of functional endocrine variation within the physiological reference range. Given the age range of the cohort (16–35 years), serum *T* values were not used to assign an age‐specific clinical diagnosis; instead, clinical testosterone deficiency (<10 nmol/L) was reported descriptively, and the primary analyses were based on within‐cohort testosterone status. In our sample, only one individual met the clinical criterion for *T* deficiency (<10 nmol/L) (Aversa & Morgentaler, 2015). Throughout the manuscript, *T* refers to serum total testosterone. To assess androgen bioavailability, the Free Androgen Index (FAI) was calculated using the equation: FAI = (Total *T*/SHBG) × 100.

### 2.4 Bone stress injuries

All participants answered a questionnaire or were interviewed by a medical doctor about their injury/illness history in the last 12 months (questions about the type of injury) and overall history of bone stress injuries (BSI) (number, type, and location of bone injuries). Since the analysis used a binary variable (any BSI yes/no), the different ascertainment modes (self‐report vs. physician interview) were not expected to materially affect the results.

### 2.5 Individual feedback after assessments

After each assessment, athletes received individual feedback from research team members or clinical staff involved in the testing procedures. The feedback consisted of an individualized summary of relevant physiological and health‐related results, including body composition, bone health, and blood marker information when available. Feedback was provided verbally or in writing as part of routine athlete support. No standardized dietary, training, or medical intervention was prescribed in the present observational analysis, and subsequent changes in athlete behavior were not controlled by the study protocol.

### 2.6 Statistical analysis

All statistical analyses were performed using IBM SPSS Statistics, version 28 (IBM Corp., Armonk, NY, USA), and R version 4.1.2 for the repeated‐measures models and sample‐size sensitivity calculations. Figures were created with GraphPad Prism (GraphPad Software, Boston, MA) and Microsoft Excel (Version 2404). The significance threshold was set at *α* = 0.05. Descriptive statistics are presented as means ± standard deviation (SD) for continuous variables. The normality of each variable was assessed using the Shapiro–Wilk test, histograms, and *Q*‐*Q* plots.

For the baseline analyses, between‐group differences were assessed using independent‐samples Student's *t*‐tests for normally distributed variables and Mann–Whitney *U* tests for nonnormally distributed variables. Effect sizes for baseline between‐group comparisons are reported as Hedges' *g* with 95% confidence intervals (CI) and unstandardized mean differences. One‐year follow‐up outcomes were analyzed using repeated‐measures analysis of variance (ANOVA), with testosterone group (low *T* vs. normal *T*) as the between‐subject factor and time (*T*1 vs. *T*2) as the within‐subject factor. The group‐by‐time interaction was the primary follow‐up test, as it directly evaluates whether 1‐year changes differed according to baseline testosterone status. Because only two time points were included, sphericity correction was not required. For descriptive clarity, absolute changes from T1 to T2 are also reported as mean ± SD.

As an additional exploratory analysis of BSI, athletes with and without a history of BSI were compared for baseline age, BMI, fat‐free mass, BMD outcomes, and endocrine markers in the baseline cohort available for the revised analyses (*n* = 46). Given the number of athletes with BSI history, these analyses were considered hypothesis‐generating; no multivariable prediction model was fitted, and *p*‐values were interpreted descriptively.

Correlation analyses were performed using Pearson's correlation coefficient for normally distributed variables and Spearman's correlation coefficient for variables with nonnormal distribution. For descriptive interpretation, absolute correlation coefficients of 0.10–0.39, 0.40–0.69, and ≥0.70 were considered weak, moderate, and strong, respectively. Multiple linear regression models were used to examine independent predictors of changes in total BMD and *Z*‐scores, adjusting for relevant covariates (e.g., baseline *T*, vitamin D, and IGF‐1, as appropriate). Analyses were performed on complete cases for each model; no data imputation was conducted.

All main outcomes and between‐group comparisons were prespecified according to the study protocol. Correlation and regression analyses were exploratory and should be considered hypothesis‐generating. Given the number of statistical tests performed, there was a risk of inflated Type I error; accordingly, exact *p*‐values are reported and interpreted alongside effect sizes and 95% CIs. No a priori sample‐size calculation was performed because this was a secondary observational analysis, and the sample size was determined by the number of eligible participants available in the cohort. Sensitivity calculations indicated that the baseline comparison (low *T n* = 12, normal *T n* = 34) had approximately 80% power to detect only large between‐group effects (Cohen's *d* ≈ 0.96), while the follow‐up comparison (low *T n* = 8, normal *T n* = 23) had approximately 80% power to detect large between‐group differences in change (Cohen's *d* ≈ 1.19). For BMD outcomes, this corresponded approximately to detectable between‐group differences in annual change of 0.022 g/cm^2^ for total BMD, 0.034 g/cm^2^ for L2–L4 BMD, and 0.023 g/cm^2^ for femur BMD.

## 3 RESULTS

Results are presented as baseline findings followed by 1‐year follow‐up findings.

### 3.1 Baseline analysis

Forty‐six endurance‐trained male athletes were included in the analysis. Of these, 12 individuals (26.1%) fell within the lowest *T* quartile and were classified as low *T*. Only one participant (2.2%) met the clinical criterion for *T* deficiency (<10 nmol/L) (Aversa & Morgentaler, 2015). The represented endurance disciplines included biathlon, cross‐country skiing, orienteering, triathlon, Nordic combined, and endurance running.

Table 1 presents the demographic, anthropometric, hormonal, and bone characteristics of male endurance athletes stratified by *T* status. Athletes in the low *T* group were older than athletes in the normal *T* group (26.6 ± 3.5 vs. 24.1 ± 3.5 years, *p* = 0.040, *g* = 0.69). No significant differences were observed between groups in height, body weight, fat‐free mass, or total body fat percentage (all *p* > 0.15; Table 1).

**TABLE 1:** Demographic, anthropometric, hormonal, metabolic, and bone characteristics of male endurance athletes stratified by testosterone status at baseline.

Variable	Normal *T* (*n* = 34)	Low *T* (*n* = 12)	Mean difference (95% CI)	*p*‐Value	Effect size (Hedges' *g*)
Age (years)	24.1 ± 3.5	26.6 ± 3.5	2.49 (−0.12 to 5.08)	0.040	0.69
Height (cm)	182.2 ± 5.4	183.8 ± 5.4	1.60 (−2.07 to 5.27)	0.385	0.29
Weight (kg)	73.4 ± 7.3	74.0 ± 7.4	0.60 (−4.33 to 5.54)	0.806	0.08
BMI	22.1 ± 2.6	21.9 ± 2.5	0.2 (−1.13 to 1.52)	0.80	0.08
VO_2max_	71.2 ± 5.9	68.1 ± 5.2	−3.04 (−6.97 to 0.88)	0.125	−0.51
FFM (kg)	67.8 ± 6.5	69.8 ± 6.7	1.97 (−2.50 to 6.43)	0.38	0.29
Lean‐mass (kg)	64.5 ± 6.3	66.4 ± 6.4	1.88 (−1.9 to 5.7)	0.38	0.29
Total body fat (%)	8.6 ± 3.7	7.4 ± 2.7	−1.97 (−7.75 to 3.82)	0.309	−0.34
Testosterone (nmol/L)	17.9 ± 3.1	11.8 ± 1.8	−6.14 (−8.04 to −4.23)	<0.001	−2.15
IGF‐1 (nmol/L)	26.0 ± 8.6	20.3 ± 4.1	−5.75 (−10.97 to −0.55)	0.031	−0.74
Cortisol (nmol/L)	447.2 ± 85.0	408.8 ± 140.0	−38.34 (−107.10 to 30.43)	0.267	−0.37
Insulin (μIU/mL)	2.2 ± 2.0	1.7 ± 2.5	−0.48 (−1.92 to 0.97)	0.511	−0.22
25 (OH)D (nmol/L)	90.7 ± 26.1	86.5 ± 11.4	−4.17 (−19.97 to 11.62)	0.597	−0.18
SHBG (nmol/L)	36.5 ± 8.4	33.6 ± 6.6	−2.87 (−8.26 to 2.52)	0.361	−0.35
FAI	52.5 ± 22.3	36.5 ± 10.1	−16.06 (−29.59 to −2.53)	<0.001	−0.79
Total BMD (g/cm^2^)	1.31 ± 0.08	1.32 ± 0.06	0.01 (−0.04 to 0.06)	0.650	0.15
Total *Z*‐score	1.24 ± 0.87	1.39 ± 0.55	0.16 (−0.38 to 0.69)	0.556	0.20
L2–L4 BMD (g/cm^2^)	1.25 ± 0.12	1.25 ± 0.14	−0.01 (−0.09 to 0.08)	0.971	0.01
L2–L4 *Z*‐score	0.31 ± 0.93	0.31 ± 1.02	0.004 (−0.64 to 0.65)	0.991	0.00
Femoral BMD (g/cm^2^)	1.21 ± 0.15	1.18 ± 0.15	−0.03 (−0.13 to 0.07)	0.522	−0.21
Femoral *Z*‐score	0.88 ± 1.09	0.69 ± 1.05	−0.19 (−0.93 to 0.55)	0.613	−0.17

*Note*: Values are presented as mean ± SD. Low *T* = lowest quartile of baseline testosterone in this cohort; Normal *T* = remaining quartiles. Mean differences are reported as low *T* minus normal *T*. *p*‐Values refer to between‐group comparisons assessed by independent *t*‐tests or Mann–Whitney *U* tests, as appropriate. Effect sizes are reported as Hedges' *g*.

Abbreviations: 25(OH)D, 25‐hydroxyvitamin D; BMD, bone mineral density; BMI, body mass index; FAI, free androgen index; FFM, fat‐free mass; IGF‐1, insulin‐like growth factor‐1; SHBG, sex hormone‐binding globulin; VO_2max_, maximal oxygen uptake.

No significant between‐group differences were observed for BMD or *Z*‐scores at any anatomical site. Specifically, total BMD was similar between the low *T* group (1.32 ± 0.06 g/cm^2^) and the normal *T* group (1.31 ± 0.08 g/cm^2^), with a mean difference of 0.01 g/cm^2^ (95% CI: −0.04 to 0.06, *p* = 0.650, *g* = 0.15). Lumbar spine BMD (−0.01 g/cm^2^, 95% CI: −0.09 to 0.08, *p* = 0.971, *g* = 0.01) and femoral neck BMD (−0.03 g/cm^2^, 95% CI: −0.13 to 0.07, *p* = 0.522, *g* = −0.21) did not differ significantly between groups. Corresponding *Z*‐scores showed no significant differences: total *Z*‐score (0.16, 95% CI: −0.38 to 0.69, *p* = 0.556, *g* = 0.20), lumbar spine *Z*‐score (0.004, 95% CI: −0.64 to 0.65, *p* = 0.991, *g* = 0.00), and femoral neck *Z*‐score (−0.19, 95% CI: −0.93 to 0.55, *p* = 0.613, *g* = −0.17).

Across all skeletal sites, most athletes had *Z*‐scores within the expected range. Overall, 5.2% of *Z*‐score values were below −1.0, and one value (0.2%) was below −2.0.

Biochemical markers are presented in Table 1. Serum IGF‐1 concentrations were lower in the low *T* group (20.3 ± 4.1 nmol/L) than in the normal *T* group (26.0 ± 8.6 nmol/L; mean difference − 5.75 nmol/L, 95% CI: −10.97 to −0.55, *p* = 0.031, *g* = −0.74). The calculated FAI was also lower in the low *T* group (mean difference − 16.06, 95% CI: −29.59 to −2.53, *p* < 0.001, g = −0.79). No significant between‐group differences were observed for serum cortisol (*p* = 0.267), insulin (*p* = 0.511), 25(OH)D (*p* = 0.597), or SHBG (*p* = 0.361).

**FIGURE 1:**
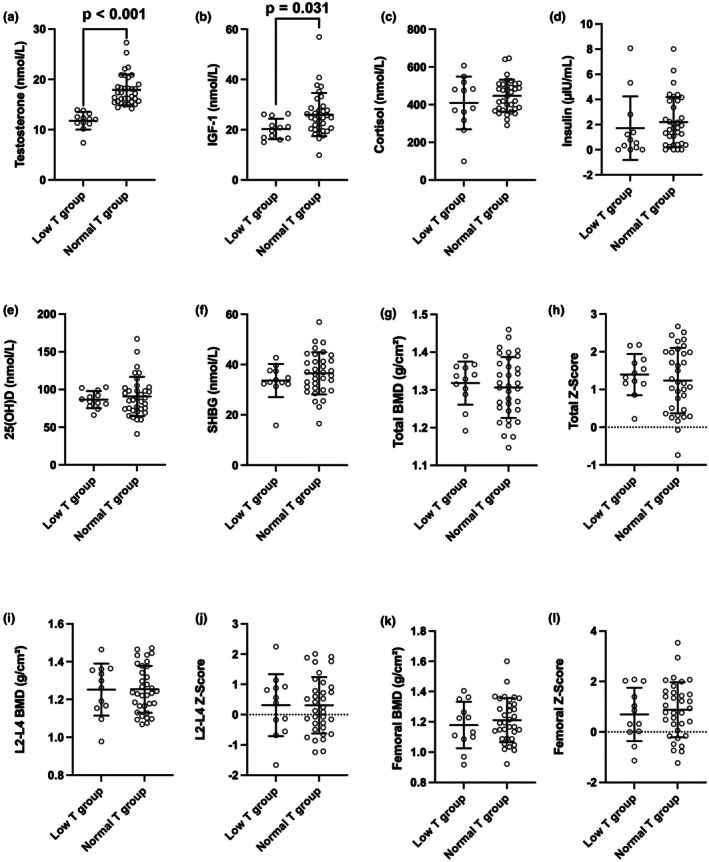
Group comparisons between male endurance athletes with low and normal testosterone concentrations for (a) serum testosterone, (b) serum IGF‐1, (c) serum cortisol, (d) serum insulin, (e) serum 25(OH)D, (f) serum SHBG, (g) total body BMD, (h) total body *Z*‐score, (i) lumbar spine (L2–L4) BMD, (j) lumbar spine *Z*‐score, (k) femoral neck BMD, and (l) femoral neck *Z*‐score. 25(OH)D, 25‐hydroxyvitamin D; BMD, bone mineral density; IGF‐1, insulin‐like growth factor‐1.

These results are shown in Table 1 and Figure 1a–l.

**FIGURE 2:**
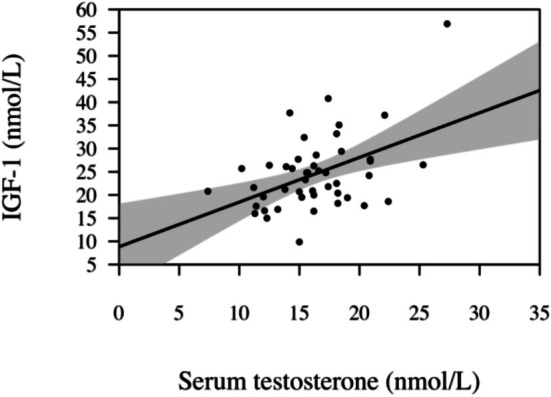
Association between serum testosterone and IGF‐1 in male endurance athletes. Scatter plot demonstrating the positive correlation between serum testosterone and insulin‐like growth factor 1 (IGF‐1) concentrations in the full cohort of male endurance athletes (*n* = 46; *ρ* = 0.355, *p* = 0.015). Each dot represents an individual participant. The solid line represents the linear regression fit, with the shaded area indicating the 95% confidence interval for visualization.

Correlation analysis at baseline revealed a positive association between *T* and IGF‐1 (*ρ* = 0.355, *p* = 0.015; Figure 2) and between FAI and IGF‐1 (*ρ* = 0.410, *p* = 0.005). Fat‐free mass was positively correlated with total body *Z*‐score (*r* = 0.309, *p* = 0.018), and fat percentage was positively correlated with lumbar spine BMD (*ρ* = 0.294, *p* = 0.025). In addition, lean mass showed positive correlations with total BMD (ρ = 0.475, *p* = 0.001), total BMC (*ρ* = 0.761, *p* < 0.001), femoral BMD (*ρ* = 0.490, *p* = 0.001), and lumbar spine BMD (*ρ* = 0.550, *p* < 0.001). Significant positive associations were also found between lean mass and site‐specific *Z*‐scores at the lumbar spine (*ρ* = 0.470, *p* = 0.001) and femur (*ρ* = 0.352, *p* = 0.016). Conversely, lean mass was negatively correlated with insulin levels (*ρ* = −0.345, *p* = 0.018). No other significant associations were observed (*p* > 0.05).

**FIGURE 3:**
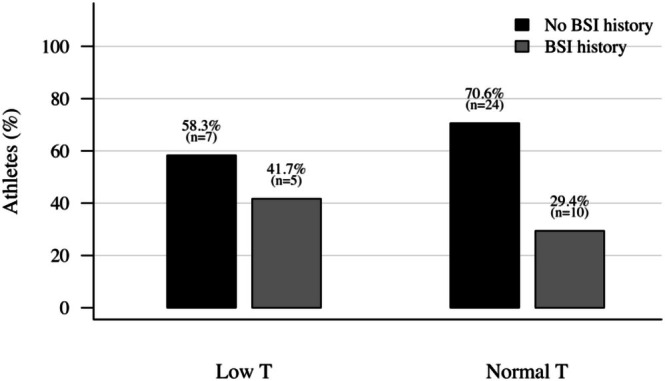
Prevalence of bone stress injuries (BSI) in male endurance athletes stratified by testosterone status. The percentage and number of athletes with (gray bars) and without (black bars) a history of BSI are shown for the low *T* group and normal *T* group. Numbers on the bars indicate the number of athletes in each category.

The proportion of athletes with a history of BSI was numerically higher in the low *T* group (41.7%; 5 of 12) than in the normal *T* group (29.4%; 10 of 34), but this difference was not statistically significant (*p* = 0.488, Fisher's exact test; see Figure 3). The odds ratio for BSI in the low *T* group compared with the normal *T* group was 1.69 (95% CI: 0.34 to 8.09).

An additional exploratory analysis in the baseline cohort (*n* = 46; BSI history *n* = 15, no BSI history *n* = 31) suggested that athletes with a BSI history had lower total‐body BMD (1.269 ± 0.067 vs. 1.329 ± 0.071 g/cm^2^, *p* = 0.010), total‐body *Z*‐score (0.92 ± 0.70 vs. 1.45 ± 0.79, *p* = 0.026), L2–L4 BMD (1.168 ± 0.099 vs. 1.294 ± 0.118 g/cm^2^, *p* < 0.001), L2–L4 *Z*‐score (−0.31 ± 0.70 vs. 0.61 ± 0.91, *p* < 0.001), and femoral BMD (1.141 ± 0.116 vs. 1.232 ± 0.152 g/cm^2^, *p* = 0.031) than athletes without BSI history. Cortisol was lower in athletes with BSI history (388.9 ± 103.2 vs. 460.5 ± 94.2 nmol/L, *p* = 0.027), and age tended to be higher (26.3 ± 4.2 vs. 24.0 ± 3.1 years, *p* = 0.066). No statistically significant differences were observed for BMI, fat‐free mass, testosterone, FAI, SHBG, IGF‐1, insulin, 25(OH)D, or femoral *Z*‐score (all *p* ≥ 0.075; Table S1).

### 3.2 Follow‐up outcomes

A total of 31 athletes completed the follow‐up assessment after 10–14 months, including 8 individuals in the baseline low *T* group and 23 in the normal *T* group. Follow‐up outcomes were analyzed using repeated‐measures ANOVA, with the group‐by‐time interaction used to test whether 1‐year changes differed according to baseline testosterone status. Results were summarized in Table 2.

**TABLE 2:** Repeated‐measures analysis of 1‐year changes in hormonal, body composition, and bone parameters.

Variable	Normal *T* Δ	Low *T* Δ	*p* group	*p* time	*p* group × time
Weight (kg)	−0.37 ± 1.64	0.24 ± 1.12	0.166	0.464	0.344
Body fat (%)	0.47 ± 2.23	−0.07 ± 1.50	0.868	0.388	0.533
Fat‐free mass (kg)	−0.56 ± 1.09	−0.38 ± 0.73	0.068	0.009	0.682
Lean mass (kg)	−0.56 ± 1.12	−0.40 ± 0.72	0.070	0.009	0.706
Testosterone (nmol/L)	−0.96 ± 2.48	3.10 ± 3.47	0.002	0.862	0.001
FAI	−5.61 ± 16.02	9.64 ± 10.79	0.754	0.538	0.019
SHBG (nmol/L)	4.45 ± 8.58	1.91 ± 7.59	0.092	0.017	0.465
IGF‐1 (nmol/L)	−1.30 ± 4.10	−1.51 ± 4.11	0.621	0.077	0.900
Cortisol (nmol/L)	−9.5 ± 100.1	−11.6 ± 53.0	0.383	0.544	0.955
Insulin (μIU/mL)	−0.43 ± 2.29	−0.30 ± 1.00	0.444	0.295	0.878
25(OH)D (nmol/L)	−2.2 ± 24.5	−3.5 ± 12.8	0.785	0.529	0.889
Total BMD (g/cm^2^)	0.0090 ± 0.0202	0.0022 ± 0.0127	0.388	0.040	0.387
Total *Z*‐score	0.167 ± 0.351	0.043 ± 0.142	0.632	0.023	0.345
L2–L4 BMD (g/cm^2^)	−0.0077 ± 0.0303	−0.0119 ± 0.0235	0.346	0.101	0.726
L2–L4 *Z*‐score	−0.024 ± 0.281	−0.087 ± 0.189	0.481	0.394	0.564
Femur BMD (g/cm^2^)	−0.0082 ± 0.0166	0.0004 ± 0.0253	0.507	0.092	0.283
Femur *Z*‐score	−0.013 ± 0.139	0.034 ± 0.192	0.416	0.971	0.458

*Note*: Values are mean ± SD change from T1 to T2. *p* group, *p* time, and *p* group × time are from repeated‐measures ANOVA with testosterone group (low *T* vs. normal *T*) as the between‐subject factor and time (*T*1 vs. *T*2) as the within‐subject factor. The group × time interaction tests whether the 1‐year change differed between groups.

Abbreviations: 25(OH)D, 25‐hydroxyvitamin D; ANOVA, analysis of variance; BMD, bone mineral density; FAI, free androgen index; FFM, fat‐free mass; IGF‐1, insulin‐like growth factor‐1.

A significant group‐by‐time interaction was observed for serum *T* (*F* (1, 29) = 12.89, *p* = 0.001). *T* increased in the low *T* group from 12.78 ± 1.62 to 15.88 ± 3.38 nmol/L (Δ = 3.10 ± 3.47 nmol/L), whereas it changed from 18.19 ± 2.72 to 17.23 ± 3.04 nmol/L in the normal *T* group (Δ = −0.96 ± 2.48 nmol/L). In the follow‐up subset, the highest baseline *T* value in the low *T* group was 14.4 nmol/L; at T2, 2 of 8 athletes in this baseline low *T* group remained at or below this cut‐off. No athlete in the follow‐up subset had a *T* concentration below 10 nmol/L at either T1 or T2.

FAI also showed a significant group‐by‐time interaction (*F* (1, 29) = 6.20, *p* = 0.019), increasing in the low *T* group and decreasing in the normal *T* group. No significant group‐by‐time interactions were observed for weight, body fat percentage, fat‐free mass, lean mass, SHBG, IGF‐1, cortisol, insulin, or 25(OH)D (all *p* ≥ 0.344), indicating that changes in these outcomes did not differ between groups.

For bone outcomes, no significant group‐by‐time interactions were observed for total BMD (*p* = 0.387), total *Z*‐score (*p* = 0.345), L2–L4 BMD (*p* = 0.726), L2–L4 *Z*‐score (*p* = 0.564), femur BMD (*p* = 0.283), or femur *Z*‐score (*p* = 0.458). There was a modest main effect of time for total BMD (*p* = 0.040) and total *Z*‐score (*p* = 0.023), but these changes were not specific to the low *T* or normal *T* group.

### 3.3 Correlations

**FIGURE 4:**
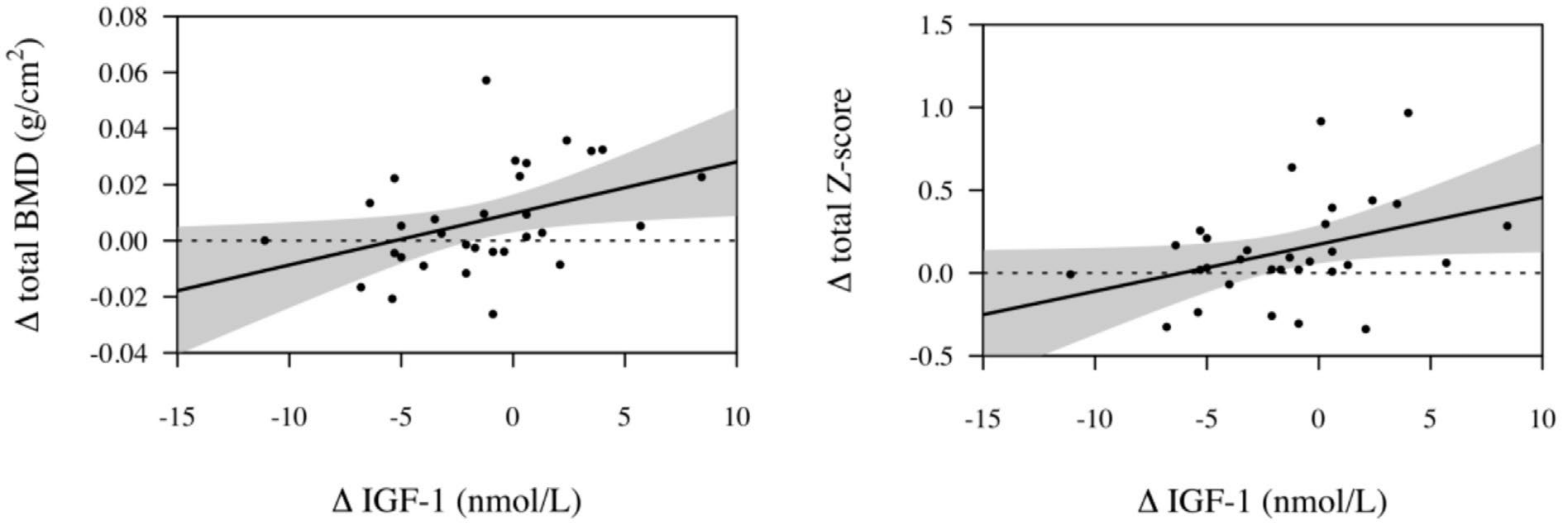
One‐year change in IGF‐1 and bone outcomes.

Correlation analyses across all participants revealed several significant associations. The increase in *T* was positively associated with a change in body weight (*ρ* = 0.370, *p* = 0.040) but not with fat percentage (*ρ* = 0.039, *p* = 0.835). In addition, change in IGF‐1 was positively correlated with change in total BMD (*r* = 0.399, *p* = 0.026) and total *Z*‐score (*r* = 0.364, *p* = 0.044; Figure 4). Furthermore, while the change in FAI was not associated with the change in total BMD (*ρ* = −0.245, *p* = 0.184), a significant positive correlation was observed between the change in FAI and the change in lumbar spine BMD (*ρ* = 0.381, *p* = 0.035). The change in lean mass was negatively associated with the change in vitamin D (*ρ* = −0.374, *p* = 0.038).

Scatter plots of ΔIGF‐1 versus Δ total body BMD (A) and Δ total body *Z*‐score (B) in athletes with baseline and 1‐year follow‐up (*n* = 31). Solid trend lines with shaded 95% CIs are shown for visualization; all statistical results are provided in the Results section.

Multiple linear regression analyses (*n* = 31) revealed that an increase in IGF‐1 was a significant independent predictor of improvements in both total BMD (*β* = 0.630, *p* = 0.01) and total *Z*‐score (*β* = 0.574, *p* = 0.017), after adjusting for baseline *T*, vitamin D, and corresponding changes. These findings were consistent with the observed positive correlations between Δ IGF‐1 and both total BMD and *Z*‐score.

## 4 DISCUSSION

The present study investigated whether being in the lowest quartile of serum total *T* is associated with differences in BMD, BSI history, and selected biochemical markers in Finnish national‐ and international‐level male endurance athletes. Overall, baseline low *T* within the clinical reference range was not associated with lower BMD, lower *Z*‐scores, or higher BSI prevalence. In the follow‐up subset, athletes classified as low *T* at baseline showed a greater increase in *T* than athletes in the normal *T* group, but 1‐year changes in BMD and *Z*‐scores did not differ between groups. These findings do not support an adverse 1‐year skeletal trajectory in athletes with low‐normal baseline *T*. Importantly, however, they should not be interpreted as evidence that chronically low *T* is without skeletal consequence. *T* was assessed at discrete standardized morning time points, and only 2 of 8 athletes classified as low *T* at baseline remained at or below the baseline low *T* cut‐off at T2. Thus, the findings are most consistent with a low‐normal and largely reversible endocrine profile in this follow‐up subset, rather than persistent clinical hypogonadism.

### 4.1 Anthropometric and body composition characteristics

Endurance sports belong to a category of athletic disciplines that emphasize leanness. A BMI < 18.5 kg/m2 is conventionally used to define underweight in adults and is considered a potential risk factor for low BMD (Tenforde et al., 2015). In our cohort, there were no statistically significant differences between groups in BMI, and no individual was below this adult underweight threshold. Other anthropometric variables did not differ between these two groups. Although age was statistically different between groups, both groups consisted of young adult males; i.e., a life stage in which age‐related biological changes in *T* are not expected to have a meaningful clinical impact (Handelsman et al., 2018). Therefore, it is unlikely that the age difference confounded the observed results in this cohort. As an indicator of aerobic performance capacity, VO_2_max was compared between groups. There was no significant difference in VO_2_max between the low *T* and normal *T* groups. Consequently, as the groups were comparable in terms of aerobic fitness, it is unlikely that differences in endocrine variables were driven by marked disparities in performance level. Correlation analyses demonstrated a positive association between fat‐free mass and total body BMD *Z*‐score, as well as between fat percentage and lumbar spine BMD. These findings are consistent with previous studies showing that both lean and healthy amounts of fat mass may contribute to bone health through mechanical loading and endocrine mechanisms (Ho‐Pham et al., 2014; Qin & Jiao, 2022; Sutter et al., 2019).

### 4.2 Blood markers

In terms of hormonal and metabolic markers, athletes with low *T* levels showed significantly lower concentrations of IGF‐1 compared to those with normal *T* levels. No differences were observed between the groups for cortisol, insulin, or 25‐hydroxyvitamin D. IGF‐1 plays a key role in bone anabolism, and its reduction may indicate an adaptive endocrine response to energetic or mechanical stress (Tahimic et al., 2013). This finding is consistent with previous reports that both IGF‐1 and *T* are sensitive to EA and training load (Hackney, 2020; McGuire et al., 2024). A significant positive correlation was also observed between *T* and IGF‐1, a relationship supported by physiological mechanisms whereby *T* stimulates IGF‐1 production (Ashton et al., 1995; Gharahdaghi et al., 2021; Veldhuis et al., 2005). Conversely, Heikura et al. (2018) did not observe a significant difference in IGF‐1 between groups based on *T* level.

With regard to insulin, our results showed no significant group differences. Similarly, Ackerman et al. (2012) reported no differences in insulin concentrations in male collegiate athletes, suggesting that insulin changes are not consistently associated with *T* status. In contrast, Heikura et al. (2018) and Koehler et al. (2016) found that male endurance athletes with low *T* or LEA had lower fasting insulin, suggesting a possible link between reduced *T*, LEA, and altered insulin sensitivity. McGuire et al. (2024) also showed that insulin levels in highly trained athletes are sensitive to changes in EA. These findings emphasize the variability of insulin responses in different study designs and populations and are consistent with the updated REDs Clinical Assessment Tool (REDs CAT2), which identifies low *T* as the primary marker for REDs in male athletes (Mountjoy et al., 2023; Popp et al., 2022). Low fasting insulin may serve as a supportive indicator when interpreted in conjunction with other clinical or hormonal signs (Mountjoy et al., 2023). Although we did not formally utilize the REDs CAT2, some endocrine patterns in our cohort may reflect early or subclinical indicators of REDs.

No group difference in morning cortisol levels was found. Both low and normal *T* athletes exhibited cortisol concentrations within normal ranges, implying that HPA axis activity was comparable between groups. This absence of elevated cortisol aligns with our broader findings and supports the interpretation that the athletes in the low *T* group were not experiencing the systemic stress or catabolic strain often associated with pathological HPG axis suppression (Anderson et al., 2018; Duclos et al., 1998).

The 1‐year follow‐up showed a significant group‐by‐time interaction for *T*, with an increase in the baseline low *T* group and a small decrease in the normal *T* group. FAI showed a similar group‐by‐time interaction, whereas SHBG showed a main effect of time but no group‐specific change. This pattern suggests that low *T* status at baseline was largely reversible in this follow‐up subset. The origin of this low‐normal *T* profile cannot be determined from the present data. It may reflect short‐term variation in training load, recovery, energy availability, or nonfunctional overreaching; it may also be compatible with an EHMC‐like adaptation in some athletes (Hackney, 2020; Hackney & Hackney, 2005; Zekarias & Shrestha, 2019). However, EHMC should not be equated simply with belonging to the lowest quartile of *T* in a cohort, because EHMC implies a more persistent adaptive‐regulatory state, ideally supported by repeated low *T* measurements and the absence of clinical or performance impairment. Conversely, LEA/REDs would be more strongly suspected when low *T* occurs together with a broader catabolic or energy‐deficient profile, such as low energy intake, weight loss, reduced IGF‐1 or insulin, impaired recovery, recurrent BSI, or other REDs‐related signs (Cupka & Sedliak, 2023; Heikura et al., 2018; McGuire et al., 2024; Mountjoy et al., 2023). In the present study, we did not measure energy availability directly and did not include bone turnover markers, and therefore cannot determine whether EHMC and LEA coexisted in individual athletes. The follow‐up measurements were conducted after the transition phase; therefore, the observed hormonal changes may partly reflect reduced training stress or behavioral adjustments after individual feedback from physiological assessments.

### 4.3 Bone health outcomes

Contrary to our hypothesis, there were no statistically significant differences in BMD or BMD *Z*‐scores at any anatomical site between the low *T* group and the normal *T* group at baseline.

Most previous studies in male endurance athletes have reported results consistent with our findings, showing no direct association between lower *T* and reduced BMD. MacDougall et al. (1992) reported no significant relationship between serum *T* and BMD across a wide range of training mileage in adult male runners. Maïmoun et al. (2003) similarly observed lower *T* in endurance athletes compared to controls, but BMD did not differ between groups. Moris et al., (2022) found that while a high proportion of collegiate male athletes exhibited low total and free *T*, BMD values were typically within the normal range and showed no significant correlation with *T* concentrations. In addition, Moore et al. (2021) showed that although many male endurance athletes display at least one component of the “male athlete triad”, most often LEA, clinically relevant negative effects on BMD were not observed. Kraus et al. (2019) and Haines et al. (2023) similarly found that while LEA and other risk factors were associated with impaired bone health, *T* levels themselves were not significantly linked to bone outcomes. Furthermore, Ackerman et al. (2012) further demonstrated that estradiol, rather than *T*, was the most significant hormonal predictor of BMD in male collegiate athletes.

An important comparison is the study by Heikura et al. (2018), in which male athletes in the low‐testosterone group had a higher prevalence of bone injuries, whereas the clearest BMD differences were observed in female athletes stratified by menstrual status rather than in male athletes stratified by testosterone status. This distinction is relevant because it suggests that low *T* in male endurance athletes may be more closely linked to injury history or broader LEA/REDs risk profiles than to consistently lower BMD per se.

The exploratory BSI analysis provides a useful qualification to the testosterone‐based comparisons. While BSI prevalence did not differ significantly by *T* group, athletes with a BSI history in the baseline cohort showed lower lumbar spine BMD and *Z*‐score, with similar patterns for total‐body BMD, total‐body *Z*‐score, and femoral BMD. This pattern suggests that injury history may reflect site‐specific skeletal vulnerability or cumulative loading and nutritional factors not captured by a single morning *T* measurement. Because BSI history was retrospective and the number of cases was small, these findings should be interpreted cautiously and require confirmation in prospective cohorts.

In the 1‐year follow‐up, there were no significant group‐by‐time interactions for total or regional BMD or *Z*‐scores. Total BMD and total *Z*‐score increased modestly over time across the follow‐up cohort, but these changes were not specific to either testosterone group. Thus, baseline low *T* status was not associated with a less favorable 1‐year bone trajectory in this sample. Nevertheless, this finding should be interpreted in relation to the reversible endocrine pattern observed at follow‐up and should not be extrapolated to athletes with persistent clinical hypogonadism or sustained REDs‐related endocrine suppression. BMD adaptations are slow, and the timing of follow‐up after the transition phase may have limited sensitivity to detect subtle or cumulative skeletal effects (Hutson et al., 2021; Popp et al., 2022; Tenforde et al., 2015).

An interesting finding was that changes in IGF‐1 were positively correlated with changes in total body BMD and corresponding age‐ and sex‐adjusted *Z*‐score over the year, and regression analysis confirmed that increases in IGF‐1 significantly predicted improvements in bone density, independent of baseline *T* or vitamin D status. This is biologically plausible, as IGF‐1 is a key anabolic factor for bone, mediating the effects of mechanical loading and hormones such as growth hormone and *T* on bone formation (Fang et al., 2023; Zhao et al., 2024). Our findings suggest that subtle changes in IGF‐1 can influence bone adaptation even in young male endurance athletes. Persistently low IGF‐1 might limit bone gains over time, though encouragingly, the low *T* group did not experience bone loss during follow‐up.

### 4.4 Strengths and limitations

This study is among the first to investigate testosterone‐bone relationships in male endurance athletes using both baseline group comparisons and 1‐year repeated assessments. Our well‐characterized cohort of national‐ to international‐level athletes was assessed for multiple aspects of bone health (including BMD at several sites and stress injury history) and a panel of hormones and metabolic markers, with standardized protocols supporting credible outcome measurement. Stratification by *T* status allowed us to explore whether subclinical low *T* is associated with different bone or metabolic profiles, and the follow‐up analysis allowed us to test whether 1‐year changes differed according to baseline *T* status.

However, the study has several limitations. First, the sample size was relatively small, particularly in the low *T* group, which limited statistical power to detect subtle differences and increased the risk of type II error (Ioannidis, 2005). Sensitivity calculations indicated that the study was powered only to detect large between‐group effects, especially in the follow‐up analysis. Therefore, small but potentially meaningful differences in BMD change cannot be excluded. Most low *T* athletes had *T* values within the normal clinical range, so our findings apply mainly to low‐normal or functional low *T*, not clinical hypogonadism (Bhasin et al., 2018). Bone stress injury history was collected retrospectively by questionnaire or physician interview and included both the preceding 12 months and lifetime history; therefore, recall bias and misclassification of injury history cannot be excluded. Second, the follow‐up duration (~10–14 months) may be too short to capture meaningful changes in bone density. Bone remodeling is a slow process, and longer‐term follow‐up or a larger sample might be required to detect cumulative effects on BMD (Tenforde et al., 2016). In addition, DXA provides areal rather than volumetric BMD and does not capture bone geometry, cortical or trabecular microarchitecture, or bone turnover; future studies should incorporate pQCT or HR‐pQCT and biochemical markers of bone turnover. Third, our hormone panel did not include estradiol (Hackney et al., 1988). Given evidence that estradiol is an important determinant of male BMD (Ackerman et al., 2012), not assessing it leaves a gap in our understanding of the hormone‐bone relationship here. It is possible that estradiol levels were well maintained even in low‐T athletes, which could explain the preserved bone outcomes, but we cannot confirm this. Fourth, although our sample consisted exclusively of Finnish male endurance athletes, there was some heterogeneity in sporting disciplines, including distance runners, orienteers, cross‐country skiers, Nordic combined athletes, triathletes, and biathletes. While this heterogeneity could contribute to greater variability in bone and hormonal outcomes, the resulting diversity in skeletal loading patterns may strengthen external validity within endurance sports. Nevertheless, our results may not be generalizable to athletes in nonweight‐bearing sports (such as cycling or swimming), sports with fundamentally different loading patterns, female athletes, or nonathletic populations. Finally, because this was an observational study, we cannot establish causation. Unmeasured confounders such as differences in training intensity, recovery strategies, or genetic factors could have influenced the outcomes. Despite these limitations, our study addresses a notable knowledge gap and provides preliminary follow‐up evidence to inform future research on male athlete bone health.

## 5 CONCLUSION

This study demonstrates that among national‐ to international‐level Finnish male endurance athletes, modest reductions in *T* within the lower end of the normal range were not associated with clinically meaningful deficits in BMD or an increased prevalence of BSIs at baseline, nor with less favorable 1‐year changes in BMD or *Z*‐scores. While lower baseline *T* was linked to reduced IGF‐1 concentrations, other metabolic and hormonal markers were largely comparable between groups, and bone outcomes were preserved over follow‐up. The observed positive relationship between changes in IGF‐1 and improvements in bone density further supports the anabolic role of IGF‐1 in skeletal adaptation in young male athletes. These findings suggest that, in this specific athletic population, bone health may be more strongly influenced by mechanical loading and broader training adaptation than by moderate, low‐normal reductions in *T* alone. However, the data do not allow us to distinguish definitively between transient LEA‐related suppression, nonfunctional overreaching, and EHMC‐like adaptation. Larger and longer studies with repeated endocrine sampling, direct assessment of energy availability, and bone turnover or microarchitectural outcomes are needed to determine whether persistent endocrine alterations, clinically low *T*, or combined REDs‐related risk factors affect bone outcomes over time.

## AUTHOR CONTRIBUTIONS

**Adam Wagner:** Conceptualization; data curation; formal analysis; methodology. **Katja Mjøsund:** Validation. **Riina Komonen:** Investigation; methodology. **Johanna K. Ihalainen:** Conceptualization; methodology; project administration; supervision.

## FUNDING INFORMATION

This study was supported by a Ministry of Education and Culture of Finland Grant (OKM/10/626/2021; OKM/78/626/2022) and with a research grant program from the International Biathlon Union (4.1.2022).

## CONFLICT OF INTEREST STATEMENT

The authors declare no conflicts of interest.

## Supporting information


**Table S1.** Exploratory comparisons by bone stress injury history in the baseline cohort.

## Data Availability

The data analyzed during the current study are available from the corresponding authors upon reasonable request.
