# Supplementary material for: Testosterone status and bone mineral density in Finnish male endurance athletes: Baseline associations and 1‐year follow‐up
Source: Physiol Rep. 2026 Jun 10;14(11):e70972. doi: 10.14814/phy2.70972 (PMC13250475; doi:10.14814/phy2.70972)
Supplement: Supplementary file 1 — Table S1. Exploratory comparisons by bone stress injury history in the baseline cohort. [file PHY2-14-e70972-s001.docx]

**Supplementary Table S1. Exploratory comparisons by bone stress injury history in the baseline cohort**

Values are mean ± SD. Positive mean differences and Hedges' g indicate higher values in athletes with a history of BSI. Analyses were conducted in the baseline cohort (BSI history n = 15; no BSI history n = 31). P-values are descriptive and were not adjusted for multiple comparisons.

| **Variable** | **BSI history (n = 15)** | **No BSI history (n = 31)** | **Mean difference** | **Hedges' g** | **Test** | **p-value** |
| --- | --- | --- | --- | --- | --- | --- |
| Age (years) | 26.3 ± 4.2 | 24.0 ± 3.1 | 2.4 | 0.66 | Welch t-test | 0.066 |
| BMI (kg/m²) | 21.7 ± 2.0 | 22.2 ± 1.7 | -0.5 | -0.25 | Welch t-test | 0.440 |
| Fat-free mass (kg) | 66.4 ± 5.8 | 69.4 ± 6.8 | -3.0 | -0.45 | Welch t-test | 0.134 |
| Testosterone (nmol/L) | 16.5 ± 4.0 | 16.2 ± 3.9 | 0.2 | 0.05 | Mann-Whitney U | 0.708 |
| FAI | 50.3 ± 33.3 | 47.4 ± 11.9 | 2.9 | 0.13 | Mann-Whitney U | 0.314 |
| SHBG (nmol/L) | 36.8 ± 8.6 | 35.2 ± 7.7 | 1.6 | 0.19 | Welch t-test | 0.551 |
| IGF-1 (nmol/L) | 24.3 ± 10.9 | 24.7 ± 6.4 | -0.4 | -0.05 | Mann-Whitney U | 0.439 |
| Cortisol (nmol/L) | 388.9 ± 103.2 | 460.5 ± 94.2 | -71.7 | -0.73 | Mann-Whitney U | 0.027 |
| Insulin (µIU/mL) | 2.27 ± 1.99 | 1.97 ± 2.21 | 0.29 | 0.13 | Mann-Whitney U | 0.354 |
| 25(OH)D (nmol/L) | 87.7 ± 24.3 | 90.5 ± 22.9 | -2.8 | -0.12 | Mann-Whitney U | 0.392 |
| Total BMD (g/cm²) | 1.269 ± 0.067 | 1.329 ± 0.071 | -0.060 | -0.84 | Welch t-test | 0.010 |
| Total Z-score | 0.92 ± 0.70 | 1.45 ± 0.79 | -0.53 | -0.69 | Mann-Whitney U | 0.026 |
| L2-L4 BMD (g/cm²) | 1.168 ± 0.099 | 1.294 ± 0.118 | -0.126 | -1.11 | Welch t-test | <0.001 |
| L2-L4 Z-score | -0.31 ± 0.70 | 0.61 ± 0.91 | -0.92 | -1.07 | Welch t-test | <0.001 |
| Femur BMD (g/cm²) | 1.141 ± 0.116 | 1.232 ± 0.152 | -0.091 | -0.63 | Welch t-test | 0.031 |
| Femur Z-score | 0.46 ± 0.88 | 1.01 ± 1.12 | -0.56 | -0.52 | Welch t-test | 0.075 |

Abbreviations: BMD, bone mineral density; BSI, bone stress injury; FAI, free androgen index; FFM, fat-free mass; IGF-1, insulin-like growth factor-1; SHBG, sex hormone-binding globulin.
